# Dietary Terpenoids in Advancing Biofilm‐Mediated Cancer Prevention: Antibiofilm Cascade, Molecular Crosstalk, and Nano‐Facilitated Functional Delivery

**DOI:** 10.1002/cbf.70281

**Published:** 2026-08-13

**Authors:** Saikat Mazumder, Aditi Dey, Debasmita Bhattacharya, Dibyajit Lahiri, Moupriya Nag, Sudhriti Maity, Navin Kumar, Soumya Pandit, Vaseem Raja, Shubham Sharma, Shashi Prakash Dwivedi, Mithul Rajeev, Juwita Ratna Dewi, Lola Sanayeva, Dilbar Azimova

**Affiliations:** ^1^ Department of Biotechnology University of Engineering and Management Kolkata West Bengal India; ^2^ Department of Food Technology Guru Nanak Institute of Technology Kolkata West Bengal India; ^3^ Department of Life Science, School of Biosciences and Technology Sharda University Greater Noida Uttar Pradesh India; ^4^ Department of Basic Science and Humanities University of Engineering and Management Kolkata West Bengal India; ^5^ Department of Biotechnology, University Centre for Research and Development Chandigarh University Mohali Punjab India; ^6^ Lloyd Institute of Engineering & Technology Greater Noida Uttar Pradesh India; ^7^ Center for Global Health Research, Saveetha Institute of Medical and Technical Sciences (SIMATS), Saveetha Medical College and Hospital Chennai Tamil Nadu India; ^8^ Postgraduate School, Environmental Sciences Universitas Brawijaya Malang East Java Indonesia; ^9^ Department of Biology Jizzakh State Рedagogical University Jizzakh Uzbekistan; ^10^ Department of Ecology National University of Uzbekistan Tashkent Uzbekistan

**Keywords:** antibiofilm, anticancer, functional food, microbes, molecular mechanism, nanotechnology, terpenoids

## Abstract

The role of microbes in cancer is gaining attention these days, especially in the context of tumor‐associated biofilms and dysbiotic microbiota. Biofilm‐producing microorganisms, such as *Fusobacterium nucleatum* and *Helicobacter pylori*, trigger oncogenic inflammation and immune evasion in tumor initiation and progression, and in the development of chemoresistance, through the activation of the NF‐κB, STAT3, and β‐catenin pathways. Dietary terpenoids are a structurally diverse group of antitumor and antibiofilm plant metabolites. Monoterpenoids, sesquiterpenoids, and triterpenoids are known to inhibit quorum sensing, the biosynthesis of extracellular polymeric substances (EPS), and the expression of biofilm‐associated virulence factors, proposing an unexplored convergence among antibiofilm and anticancer mechanisms. Importantly, the biofilm structure (thickness, developmental stage, EPS density) affects the efficacy of terpenoids, affecting diffusion, microbial persistence, and therapeutic susceptibility. The quorum‐sensing disruption is more effective in the early stages of biofilms, while high concentrations of EPS in mature, thick biofilms will require more penetration to disrupt quorum sensing. Innovative functional food matrices, including nano‐enabled delivery systems, are emerging strategies to improve bioavailability and microbiome modulation of terpenoids. Furthermore, nano‐formulations allow better penetration in dense biofilm matrices, protect terpenoids from early degradation, and allow prolonged and focused drug release in the tumor microenvironment associated with biofilms. Combining precision nutrition with microbiome‐informed dietary strategies can be used to prevent and treat cancer. The present review combines studies linking biofilm‐driven carcinogenesis with terpenoid‐mediated antibiofilm–anticancer pathways and nano‐mediated functional delivery and biofilm penetration, including highlighting the potential for microbiome modulation in cancer therapy.

## Summary

Cancers are driven more and more by tumor‐associated microbial biofilms, which induce chronic inflammation, immune system disorders, and chemoresistance. The current review proposes the novel concept of the antibiofilm‐anticancer axis and the role of dietary terpenoids as multifunctional agents with the capability to disrupt quorum sensing and the integrity of the extracellular polymeric matrix of biofilms, as well as quorum‐sensing inhibition, the NF‐κB/STAT3 signaling pathways, epithelial–mesenchymal transition, and tumor immunity. The review proposes a novel and translational platform based on the chemistry of terpenoids, the biology of tumor–microbiome interaction, and nano‐enabled functional food delivery systems for the prevention of cancer.

## 1 Introduction

Cancer is one of the leading causes of death globally [1]. Genetics, tumor microenvironment (TME), and immune systems have long been researched. But recent discoveries revealed that cancer is caused and augmented by biofilm‐forming microbes [2]. Microbial biofilm can lead to cancer cases in 11%–16% of cases of cancer worldwide, including cases of gastrointestinal cancer and colon cancer [3, 4, 5]. Biofilms are complex, structured microbial communities that grow on surfaces and are surrounded by a self‐produced EPS, which is primarily comprised of polysaccharides, proteins, extracellular DNA (eDNA), lipids, and various biomolecules. Biofilms have a complex 3D structure, where EPS serves as a selective barrier for penetration of antimicrobial agents, immune mediators, and environmental stresses, and supports nutrient retention and cell‐to‐cell communication [6]. In this controlled space, there is a high metabolic and physiological diversity of microorganisms forming microenvironments with oxygen, pH, and nutrient gradients. Consequently, subpopulations of persister or slow‐growing cells live in concert with metabolically active cells, all of which are responsible for the incredible tolerance of biofilms to antimicrobial therapy [7]. Interestingly, the inherent phenotype of the microorganisms within the biofilms is distinct from that which exists in planktonic form, meaning that altered gene expression, quorum‐sensing activity, virulence factor production, stress responses, and antimicrobial susceptibility are all present. The above unique biological properties render biofilms as long‐lasting sources of chronic infection and as a significant and emerging environmental cause of inflammation‐associated carcinogenesis [8]. Chronic infections are associated with biofilm matrixed in EPS and adhering to living and/or inanimate material [2]. Colorectal, breast, pancreatic, and lung cancers are associated with bacterial biofilms [9]. These are identified in neoplasm tissues, stromal cells, and metastases in distant organs. *Helicobacter pylori* and *Salmonella typhi*, *Fusobacterium nucleatum,* can form biofilms and are closely associated with oncogenesis [10].

While *F. nucleatum* and *H. pylori* have been widely studied in biofilm‐driven malignancies, there is a growing number of other microbes, such as *Bacteroides fragilis* is involved in colorectal cancer (CRC) by producing *B. fragilis* toxin, compromising the integrity of the epithelial barrier, turning on STAT3 and NF‐κB signaling pathways, and inducing chronic inflammation [11]. The genotoxin colibactin produced by *Escherichia coli* (*pks* ^*+*^) leads to DNA double‐strand breaks, genomic instability, and mutations associated with the development of CRC [12]. Further, *Porphyromonas gingivalis* has been associated with oral squamous cell cancer and pancreatic cancer via biofilm formation, immunological dysfunction, and epithelial–mesenchymal transition. Furthermore, *Streptococcus gallolyticus* subsp. *gallolyticus* is one of the important colorectal neoplasia biomarkers. Hence, cancer‐associated biofilms involve a broader community of microbes [13, 14].

Among all, *F. nucleatum* and *H. pylori* biofilm‐mediated CRC and gastric cancer (GC) are the prominent ones. Studies concerning the human microbiome have shown the presence of invasive biofilms, especially with high levels of *F. nucleatum*, in colorectal malignancies [15]. Moreover, Biofilm‐related *F. nucleatum* has been shown to cause malignancies by activating adhesins, E‐cadherin/‐catenin, Toll receptor, and others, in the impairment of anticancer immunity [16, 17]. Furthermore, biofilms have been linked to stomach cancers [18]. An emerging concept in gastrointestinal oncology is that, instead of monomicrobial, polymicrobial biofilms promote carcinogenesis. The most typical biofilms associated with tumors include *F. nucleatum*, *E. coli* (*pks* ^*+*^), enterotoxigenic *B. fragilis*, *Peptostreptococcus anaerobius*, and other commensal or opportunistic organisms that act in a synergistic manner [19, 20]. In polymicrobial communities, there is increased metabolic cooperation, quorum‐sensing communication, horizontal gene transfer, and production of extracellular matrix, which leads to increased ecological stability, virulence, and resistance to host defenses when compared to a single‐species biofilm. These microbial interactions exacerbate epithelial barrier dysfunction, chronic inflammation, genotoxicity, immune suppression, and tumor‐promoting signaling pathways, thus promoting gastrointestinal carcinogenesis. Therefore, it is crucial to understand the collective behavior of polymicrobial biofilms for the development of effective microbiome‐targeted anticancer interventions [21, 22].

Biofilms promote cancer through several mechanisms that induce inflammation [9], regulate and inhibit the host immune system [4], release toxins that work as carcinogens [23], and alter the host metabolic pathways [24] to promote the growth of cancer cells [25]. New evidence suggests that the bacteria within the tumor microenvironment (TME), called the tumor microbiota (TM), play an important role in the development of cancer [24].

It is also crucial to differentiate between biofilm‐associated tolerance and traditional antimicrobial resistance (AMR). In general, the emergence of AMR occurs stably through the presence of resistance genes, target site mutations, enzymatic drug degradation, or active efflux systems that decrease the susceptibility of the microorganism without regard to its lifestyle [26, 27]. Biofilm‐associated tolerance, on the other hand, is mostly reversible phenotypic changes caused by limited freedom of the antimicrobial agent to penetrate the EPS matrix, decreased metabolic activities, nutrient depletion, and the generation of persister cells and stress‐responsive physiological states. Therefore, an antimicrobial‐resistant subpopulation of genetically susceptible microorganisms might exist in planktonic culture that subsequently develops AMR when cultured in older biofilms. This separation of biofilm‐mediated tolerance from genetically acquired resistance is especially important in cancer‐associated biofilms where biofilm‐mediated tolerance is associated with persistent infection, chronic inflammation, immune system evasion, and decreased therapeutic effectiveness, which does not necessarily require genetic acquisition of resistance [8, 28, 29]. The medicines for the treatment of such malignancies are sometimes toxic and require finding alternative natural substances [30]. Further, the reason behind the escalating interest in natural chemicals is their ability to negatively affect multiple cancer hallmarks and are usually less toxic relative to traditional chemotherapeutic agents [31]. Plant and food‐sourced terpenoids are being recognized as promising anticancer agents due to their multiple biological properties, such as anti‐inflammatory and antioxidant properties [32]. Recent studies show that these agents possess anticancer properties such as inhibiting the growth of cancer cells, inducing apoptosis, and inhibiting metastases. Agents such as thymoquinone, eugenol, and geraniol possess promising anticancer properties against multiple forms of cancer [31]. β‐Elemene, limonene, ursolic acid, and other terpenoids have shown the property of modulating several pathways, viz., the NF‐κB, PI3/AKT, and MAPK pathways [33]. Further, eugenol affects S‐phase arrest by modulating the levels of the key regulatory proteins, one of which is E2F1. Terpenes also boost the expression of the pro‐apoptotic protein, Bax, while suppressing the expression of the antiapoptotic protein, Bcl‐2, thereby triggering apoptotic pathways. This approach is more favorable for the demolition of tumor cells, thereby overcoming the resistance put up against standard chemotherapy. Terpenes may act directly on cancer cells as well as the TME. They may also suppress the pro‐inflammatory cytokines and growth factors that promote tumor growth and metastasis. Some compounds, for example, linalool, suppress NF‐κB and COX‐2 activation and expression, which are linked to inflammation and cancer [34].

On the other hand, the literature indicates the antipathogenic action of terpenoids, namely, quorum sensing, the reduction of the extracellular polymer substance matrix, the disruption of the cell membrane, and the reduction of the expression of microbial genes in biofilms, which could be implicated in the role played by the microbiome in the induction of carcinogenesis in tumors, respectively [35, 36]. One of these translational opportunities is tumor–bacteria interaction, specifically the interaction between tumor cells and bacteria mediated through biofilm. For instance, *F. nucleatum* bacteria mediated through biofilm interaction in CRC have been attributed to resistance to anticancer drugs through E‐cadherin and β‐catenin interaction [15, 17]. In addition to that, microbial signaling enhances inflammation through nuclear factor kappa‐light‐chain‐enhancer of activated B cells (NF‐κB)/signal transducer and activator of transcription‐3 (STAT3) interaction [16]. Nano‐delivery platforms have been developed for lipophilic terpenoids to improve solubility, duration of biofilm interaction, and release characteristics for interacting with biofilm and microbiota. Alternatively, precision nutrition food platforms have been developed for specific types of cancers based on microbiota–biofilm interaction that is cancer‐type specific [37, 38].

In spite of these prevalent processes, however, the antibiofilm–anticancer effects of dietary terpenoids have remained insufficiently explored within the translational perspective of substantial anticancer research. Hence, this present review represents terpenoids' mechanisms of action that are integrated with biofilm‐mediated tumorigenesis, terpenoid antibiofilm action, anticancer signal transduction, and nano‐structured/functional food delivery systems for the GI tract. Finally, integrating a food‐science and microbiome perspective on terpenoids presents the conceptual framework of a dietary antibiofilm–anticancer axis, which offers a novel contribution to the fields of oncology, food, microbiome, and functional nutrition.

## 2 Microbial Biofilm and Cancer Evolution: Mechanisms and Signaling Cascade

Biofilms, an assembly of microorganisms surrounded by a protective matrix known as EPS, have been associated with many diseases, such as cancers [9, 39]. Biofilm formation is a dynamic and highly regulated process, with five stages: initial reversible attachment, irreversible adhesion, microcolony formation, maturation, and dispersion. Each developmental stage has a distinct biological profile that significantly impacts both disease potential and antimicrobial susceptibility, directly affecting the therapeutic response to the disease. In early stages of attachment and microcolony formation, bacterial cells have a high metabolic state, little EPS deposition, and more reliance on quorum sensing for community formation [40]. Consequently, these immature biofilms are still relatively vulnerable to antimicrobial agents such as dietary terpenoids that target quorum sensing, EPS initial biosynthesis, and membrane integrity [41]. In contrast, mature biofilms have a well‐defined three‐dimensional structure comprised of abundant polysaccharides, extracellular DNA, proteins, and lipid components, all of which contribute to the formation of a protective matrix. Mature biofilms also develop gradients of oxygen and nutrients, slow‐growing or dormant persister cells, increased horizontal gene transfer, and increased stress‐response mechanisms, all of which significantly decrease the susceptibility to antimicrobials [25, 42]. Thus, the antibiofilm activity of terpenoids is strongly dependent on the stage of biofilm development, with quorum‐sensing inhibition and prevention of biofilm formation being much more effective at early stages of biofilm development, while mature biofilms require higher concentrations or longer exposure times or special delivery systems to penetrate the dense EPS matrix [17, 43].

**Figure 1:**
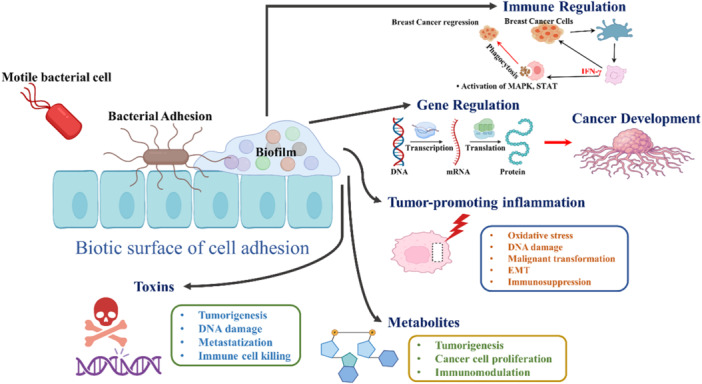
Biofilm in cancer development.

After the maturity of biofilms, the dispersion of microbes takes place, and the microbes get attached to a new surface or site in the host and thereby develop biofilm‐related infection metastasis [39]. In liver and colon cancers, a specific type of bacteria, gallolytic bacteria, related to each species, develops biofilms [44]. DNA damage, cell cycle inhibition, and chronic inflammation, which trigger cancers, can be carried out by most bacteria, such as *S. typhi* and *H. pylori*. They have been shown to influence immunity, metabolism, and inflammation [45]. Cancer, microbiomes, and biofilms have been shown to influence host cell homeostasis, immunity, and metabolism. Microbiomes in tissues, like gut bacteria, have been found to alter host processes, which trigger chronic inflammation to cause cancer. Biofilms, microbiomes, and immunity have been shown to impact defense metabolism, immunosupport, and carcinogenesis. Biofilms promote immunity, impeding it, by which microorganisms survive [46]. The right side of the colon is important in carcinogenesis. Stability, immunity, and immunology have been shown to accelerate cancers and malignancy [47] (Figure 1).

The microbes involved in the formation of tumors affect the signaling pathway involved in the progression of cancer. Cellular motility, growth, survival, and metabolism are affected by signaling pathways in cancerous and normal cells. Signal transduction in cancer involves the use of microbes that develop tumors (Table 1). These microbes affect the signaling pathway of cancer development. Survival, growth, proliferation, inflammation, invasion, and the regulation of stem cells involve all the signaling pathways (Table 1) [48]. *H. pylori* injects the virulence factor, cytotoxin‐associated gene A (CagA), into the epithelial cells of the stomach, leading to neoplastic change (Table 1) [25]. It was later revealed that *H. pylori* does not induce GC; rather, it alters the stomach microbiome by reducing the ability of other bacteria to colonize, thus forming a carcinogenic environment. In addition, it was found that biofilm production is present in breast implant‐associated anaplastic large‐cell lymphoma, suggesting the role of biofilm in the causation of these blood cancers [49]. Recent studies have revealed the significance of biofilm production in the causation of CRC, with polyps being biofilm precursors [39].

**Table 1:** Signaling pathway, potential microbes, and molecular mechanism in biofilm‐mediated cancer development.

Signaling pathway	Key microbial activators	Molecular mechanism	Cancer type involved	References
WNT/β‐catenin	*H. pylori* (CagA), *Salmonella* (AvrA), *B. fragilis* (BFT), *F. nucleatum* (FadA), HBV (HBx protein)	E‐cadherin disruption, β‐catenin phosphorylation/acetylation, TCF/β‐catenin complex formation, HBx inhibits p53	Colorectal cancer (CRC), gastric cancer (GC), hepatocellular carcinoma (HCC), breast cancer	[50, 51, 52, 53]
PI3K/AKT/mTOR signaling	*Streptococcus, Veillonella*, HIV (gp120), HBV (HBx), EBV (LMP1), *P. anaerobius*	PI3K upregulation in lower airways; viral protein‐mediated activation	Lung cancer, breast cancer, pancreatic cancer, HBV‐related cancers, HIV‐associated malignancies	[53, 54, 55]
ERK (MAPK)	*F. nucleatum, P. gingivalis* (gingipain)	Toll‐like receptor 4 paired with myeloid differentiation primary‐response protein 88 (TLR4–MYD88)–NF‐κB pathway activation, MAPK–extracellular signal‐regulated kinase (ERK) cascade, promotes cell proliferation via phosphorylation	CRC, CRC liver metastasis	[53, 56, 57]
Dectin‐1–CARD9 signaling	*Aspergillus sydowii* (β‐glucan), fungal TM	Fungal β‐glucan recognition; dectin‐1 engagement; caspase‐recruitment domain 9 (CARD9) pathway activation	Lung adenocarcinoma (LUAD)	[53, 58, 59, 60]
Aryl hydrocarbon receptor (AhR)	*Lactobacillus reuteri* (indole), *Pseudomonas* (indole derivatives), tryptophan metabolizers	Indole and I3A metabolite production; AhR activation in myeloid cells and CD8 T cells	Pancreatic ductal adenocarcinoma (PDAC), melanoma, CRC, bladder cancer	[53, 61, 62, 63]
cGAS–STING signaling	*Akkermansia muciniphila* (Akk), LGG (*Lactobacillus rhamnosus* GG), *P. gingivalis*,	cGAS recognition of double‐stranded DNA; bacterial CDN production (c‐di‐AMP, c‐di‐GMP); OMV‐mediated STING activation; mtDNA release	CRC, melanoma, lung cancer, breast cancer	[27, 64, 65, 66]
NF‐κB signaling	*F. nucleatum* (Fap2, FadA, RadD)	Fap2–CD147 binding activates PI3K–AKT–NF‐κB; FadA–E‐cadherin interaction; RadD–CD147 axis; BFT‐induced MAPK activation	Colorectal cancer, gastric cancer, oral squamous cell carcinoma	[65, 67, 68]

**Figure 2:**
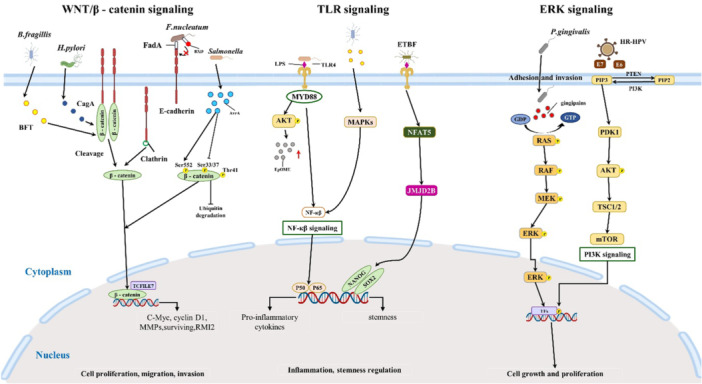
Biofilm‐mediated signaling cascade in cancer development.

Studies have shown that cancer signaling processes in host tissues may be impacted by biofilms in several ways. Biofilm‐induced chronic inflammation and biofilm‐related proinflammatory cytokines fuel oncogenic processes involving NF‐κB, STAT3, and mitogen‐activated protein kinase (MAPK)/extracellular signal‐regulated kinase (ERK), driving preneoplastic cell proliferation and survival (Figure 2). Biofilm‐induced bacteria and some of their virulence factors activate bodily receptors like integrins and TLRs, causing activation of the phosphoinositide 3‐kinase (PI3K)/protein kinase B (Akt)/mechanistic target of rapamycin (mTOR) and Wnt/β‐catenin pathways, which can lead to cancer, cell movement, and suppression of the immune system (Figure 2). Biofilm‐related oxidative stress and the disturbance of the cellular oxygen and nitrogen species, which cause DNA damage, also activate cellular signaling pathways, including oncogene suppression (Figure 2). These combined pathways of biofilm and cellular pathways fuel inflammation‐related cancer, immune system evasion, and resistance to chemotherapy [18, 69].

One of the emerging concepts in cancer microbiology is the resemblance between tumor‐associated biofilms and the TME. Both systems have high levels of hypoxia, acidity, nutrient limitation, oxidative stress, and significant spatial heterogeneity [39]. Mature biofilms contain metabolically distinct microorganisms with low oxygen levels and limited nutrients, with high levels of persister cells that are highly resistant to antimicrobials. Likewise, the hypoxia‐induced pathways that are activated in hypoxic solid tumors (hypoxia‐inducible factor 1‐alpha (HIF‐1α), NF‐κB, STAT3, and PI3K/AKT) promote metabolic reprogramming, angiogenesis, EMT, and resistance to chemotherapy [7, 70]. Under these common physiological conditions, reciprocal interactions are created between the tumor cells and the resident microorganisms within a biofilm, supporting chronic inflammation, immune suppression, and persistence of microbes. Importantly, biological activities of dietary terpenoids may also be affected by environmental factors within the TME. Changes in oxygen supply, local pH, nutrient gradients, and others can impact terpenoid diffusion, chemical stability, microbial uptake, and intracellular signaling responses [45, 71]. However, to comprehend the terpenoids' counterbalance mechanisms of the biofilm, the microphysicochemical environment of the tumor should be taken into account along with microbial composition.

## 3 Biological Activities of Terpenoids in Biofilm‐Mediated Cancer Therapy: Commencing Food Matrix‐Driven Source to Antibiofilm and Cancer Inhibition

### 3.1 Food Matrix‐Driven Source and Signaling Crosstalk

Terpenoids are organic compounds that can be derived from diverse plant and food sources, and have received significant attention owing to their antitumor properties. They have a broad spectrum of biological actions like inducing apoptosis, restraining metastasis, and altering various signal transduction pathways, which can make them useful in cancer treatment. They have the potential to increase the effectiveness of current strategies to combat cancer and are useful in developing a multipronged approach to cancer treatment [72]. Based on the studies, one of the most favorable aspects of terpenoids is their ability to enhance the effects of synthetic chemotherapeutic agents. However, certain terpenoids can increase the cytotoxic effects of certain chemotherapeutic agents like doxorubicin and paclitaxel, which is especially beneficial when the tumors are chemoresistant. In these cases, terpenoids can circumvent these resistance obstacles by acting on different pathways responsible for the growth and survival of the tumor. Considering their ability to enhance the uptake of chemotherapeutic agents by cancerous cells and their ability to modulate drug metabolism, terpenoids can be used to devise more effective treatment plans [73, 74]. Terpenoids can affect the microbiome–TME, which is crucial in cancer development and metastasis. Also of interest, natural compounds like curcumin and resveratrol have been documented to reduce the secretion of some pro‐inflammatory cytokines and the inflammatory response related to the production of growth factors that increase tumor angiogenesis and promote tumor progression. Terpenoids can alter the carcinogenic microenvironment in the host and lead to metastasis or make the microenvironment less supportive for malignancy. This multifaceted approach of terpenoids not only suppresses or spreads of cancer cells but also undermines the environment's need for cancer cell growth [75].

Research shows the role of microbial biofilms formed from *F. nucleatum* in colon cancer through the NF‐κB, β‐catenin, MAPK/ERK, and STAT3 pathways (Table 2) [15, 76]. Further, it has also been observed that terpenoids can inhibit the actions of diverse carcinogenesis pathways, including PI3K, AKT, or mTOR pathways, which are often hyperactivated in various cancers. By downregulating these pathways, terpenoids counteract cancer cells and positively induce cell cycle arrest and apoptosis. Moreover, they can modulate many important pathways, including the MAPK pathway, thereby increasing their anticancer activity [77]. This evidence explains the relationship between certain biofilms and colon tumors and has shown that biofilms are found in the tumor mucosa and are related to the inflammation of the tumor and the proliferation of the epithelial tissues [78]. Food‐derived terpenoids have shown the ability to modify the inflammation pathways of the affected tissues and also disrupt biofilm formation and reduce biofilm‐associated resistance to chemotherapy (Table 2). Terpenoids have shown the ability to modify the inflammation pathways of the affected tissues and also disrupt biofilm formation and reduce biofilm‐associated resistance to chemotherapy. Further, Table 2, terpenoids have been related to the type of cancer, the organisms that are responsible for the formation of biofilms, and the molecular pathways that are affected (Table 2). This relationship brings together terpenoids, biofilms, and cancer (Table 2).

**Table 2:** Food/plant‐derived terpenoids in biofilm‐mediated cancer prevention: microbial cross‐linkages and signaling crosstalk.

Class	Type of terpenoids	Food/plant source	Antibiofilm activity against	Anticancer activity	Key host mechanism/signaling involved	References
Monoterpenoid	Thymol	Oregano, *Thymus vulgaris*	*H. pylori*, *F. nucleatum*	Colorectal and stomach cancer	↓NF‐κB ROS↑ and anti‐inflammation	[79]
	d‐Limonene	Citrus fruit peels	*F. nucleatum*, *E. coli* (pks*)	Colorectal cancer	Induce apoptosis and PI3K/Akt↓,	[80]
	Thymoquinone	*Nigella sativa*	*F. nucleatum*, enterotoxigenic *B. fragilis*	Breast and colorectal cancer	NF‐κB↓, p53↑, and induce apoptosis	[79]
	Auraptene	Citrus fruit peels	*F. nucleatum* (colorectal cancer biofilm)	Colon cancer	ERK/MAPK/mTOR↓	[79]
	Geraniol	Citrus fruits, berries, herbs, carrots, ginger	*F. nucleatum, E. coli* (pks*)	Colorectal and prostate cancer	ERK↓	[81]
Diterpenoid	Triptolide	*Tripterygium wilfordii*	*H. pylori*	Gastric and pancreatic cancer	NF‐κB/Akt/mTOR↓	[65]
	Crocetin	*Crocus sativus, Gardenia jasminoides*	*F. nucleatum* (colorectal cancer biofilm)	Breast and colorectal cancer	PI3K/Akt↓ and oxidative stress↓	[82]
Triterpenoid	Betulinic acid	Birch bark, *Ziziphus jujuba, Diospyros spp*, and papayas	*F. nucleatum*, *E. coli* (colorectal cancer biofilm)	Colorectal cancer	Induces apoptosis in Mitochondria	[79]
	Ursolic acid	Apple peel, herbs, olives, grapes, and berries	*F. nucleatum*, *B. fragilis*	Breast and colorectal cancer	NF‐κB/STAT3↓	[83]
	Lupeol	Fruits such as strawberries, mangoes, olives, grapes, cucumbers, and tomatoes	*H. pylori*	Prostate and gastric cancer	NF‐κB↓,	[80]
		Vegetables such as cabbage, tomato, carrots, and peas				
Sesquiterpenoid	β‐Elemene	*Artemisia annua*	*F. nucleatum*	Colorectal and lung cancer	PI3K/Akt/MAPK↓	[79]
	Artemisinin	*Curcuma* spp.	*H. pylori*	Colorectal and gastric cancer	mitochondrial stress	[82]
Phenylpropanoid	Eugenol	Clove	*H. pylori*; *P. gingivalis*	Oral and gastric cancer	NF‐κB↓	[82]

*↑ = upregulation, ↓ = downregulation.

Though there is a significant amount of evidence that terpenoids have antibiofilm and anticancer properties, there are still many inconsistencies in experimental design. Most antibiofilm studies employ in vitro biofilm systems that represent single‐species biofilms, while biofilm systems associated with tumors are polymicrobial and dynamically interact with immune and stromal components. Likewise, numerous anticancer studies have been conducted using high doses of exposure in monocultures of cancer cell lines without considering the context of the microbiome associated with cancer. Thus, there are still difficulties in the direct translation of current findings to clinical oncology. One further drawback is the absence of consistent potency data comparisons of the terpenoids for their activity in inhibiting biofilm, suppressing quorum sensing, inducing apoptosis, and modulating immunity. Mechanistic relationships between the disruption of biofilms and anticancer efficacy should be explored in future investigations by combining microbiome‐informed tumor models, organoids, and coculture systems.

### 3.2 Terpenoids as an Antibiofilm Agent: Downregulation of Biofilm–Cancer Axis

**Figure 3:**
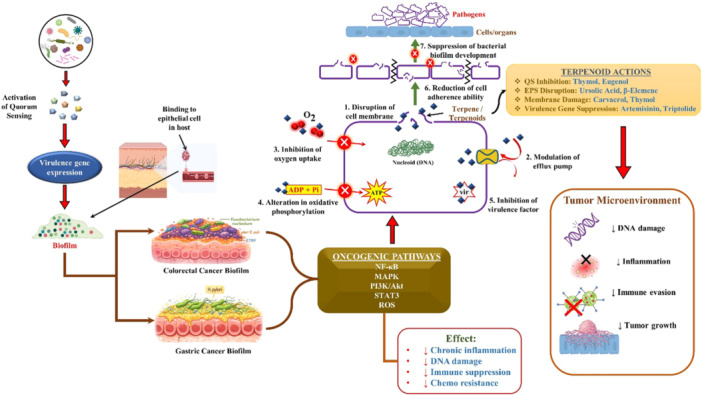
Terpenoids in the inhibition of biofilm and virulence factors in the host.

Quorum sensing (QS) controls the expression of certain genes related to biofilm maturity, aggressiveness, and coordination of microorganisms. Terpenoids, such as thymol, eugenol, geraniol, and d‐limonene, reduce biofilm formation and virulence expression by interfering with the QS signal and transcriptional regulators in Gram‐negative biofilms and polymicrobial biofilms [84]. In CRC‐related biofilms, which are comprised mainly of *F. nucleatum* and *E. coli* (pks* strain), there is a reduction in the inflammation induced by microbes, which leads to a weakened activation of NF‐κB and MAPK pathways by disruption of QS (Figure 3). This also reduces the inflammatory responses that have a tumorigenic effect [85]. The EPS matrix of biofilms protects the immune system and antimicrobials. In addition to its structural role, EPS is also a very selective physicochemical barrier regulating molecular transport in biofilms. The negatively charged polysaccharides, extracellular DNA, proteins, and divalent cation‐containing network form a diffusion‐limiting network that allows the selective restriction of penetration of antimicrobial agents based on their molecular size, charge, hydrophobicity, and binding affinity [86]. Additionally, EPS adsorbs antimicrobial compounds and binds metal ions like Ca^2+^, Mg^2+^, and Fe^3+^, which stabilizes the architecture of biofilm and decreases the availability of antimicrobials. Creating steep diffusion gradients in the EPS matrix creates a metabolically diverse microenvironment of dormant persister cells with an amazing tolerance for antimicrobial treatment [86, 87, 88]. Thus, the disruption of EPS is not only matrix degradation, but it also allows better penetration of the antimicrobial agents, better access to immune cells, a disruption of the mechanical integrity of the biofilm, and resensitization of microbials embedded in the biofilm. In the context of tumor‐associated biofilms, the properties are especially significant as the EPS is dense enough to cause chronic inflammation and immune evasion and to perpetuate oncogenic signaling pathways [25].

In addition to EPS‐induced protection, mature biofilms contain metabolically dormant persister cells which contribute to biofilm‐specific antimicrobial tolerance. Persister cells survive by entering into reversible metabolic dormancy, which involves decreased adenosine triphosphate (ATP) production, increased activity of the stringent response and toxin–antitoxins, oxidative stress adaptation, and reduced antibiotic target activity, rather than through genetically resistant bacteria. Therefore, the antimicrobials used traditionally are ineffective at killing them, and biofilm reinfection is likely to occur [8, 29]. However, terpenoids may help counteract persister‐associated tolerance indirectly by disrupting EPS integrity, elevating the permeability of the membranes, reducing quorum sensing, and inhibiting virulence pathways to improve antimicrobial penetration [43]. However, research is scarce in this area, and more studies are highly needed.

Terpenoids reduce EPS by destroying polysaccharide synthesis and the interactions of proteins and lipids, as well as the cohesion of the matrix (Figure 3) [35]. Examples of terpenoids that highly reduce EPS and biofilm structure are triterpenoids, such as ursolic acid, betulinic acid, and lupeol, and sesquiterpenoids, such as β‐elemene. In polymicrobial CRC biofilms, EPS destabilization is particularly relevant, since this biofilm's dense matrix promotes epithelial invasion and extended inflammation [35]. Many terpenoids have shown strong and swift biofilm activities through interaction with the bacterial cell membrane, which caused increased permeability and cellular metabolism disturbance because of ion leakage [35]. There are some monoterpenoids that enter the lipids of the bacterial cell wall, such as eugenol, carvacrol, and thymol, and might damage the cell membrane and ATP production [89]. In the context of biofilm‐associated GCs, a collapse in cellular metabolism due to the bacterial membranes would make the *H. pylori* biofilm more vulnerable to oxidative stress and decrease the signaling for ROS‐induced DNA damage in the epithelial cells (Figure 3) [89].

The effectiveness of the disruption of the EPS architecture is effective, but the effectiveness of this depends on the maturity of the biofilm, the species of microbes, and the duration of treatment. EPS is a dynamic matrix that can be quickly regenerated by re‐secretion of polysaccharides and quorum‐sensing‐mediated matrix synthesis upon the removal of antimicrobial stress [90]. Thus, while it is certain that the transient disruption of EPS will increase the likelihood of the bacterial cells getting exposed to host immunity and antimicrobial agents, it is not known that it will completely prevent the establishment of biofilm [40]. Long‐term control of the biofilm is therefore likely to require sustained inhibition of quorum sensing, EPS biosynthetic pathways, and virulence gene expression. However, repeated administration or controlled‐release nanoformulations seem to be able to extend the local terpenoid availability, delay the regeneration of the EPS, and to decrease the recurrence of even biofilms. Temporal kinetic studies of EPS recovery from terpenoid‐treated tumor‐associated polymicrobial biofilms, however, remain limited and are an important area that needs further investigation [40, 43].

Besides physically disrupting biofilms, terpenoids inhibit the expression of genes associated with the organism's virulence and biofilm formation, which include genes encoding mechanisms involved in adhesion, toxins, and immune evasion, among others [35]. Sesquiterpenoids and diterpenoids, including artemisinin compounds and triptolide, lower the expression of virulence factor genes associated with NF‐κB, STAT3, and MAPK signaling and inhibit biofilm‐mediated inflammation. They play a role in GC, where the virulence of *H. pylori* biofilm perpetuates epithelial‐to‐mesenchymal transition and immune evasion [35].

Further, biofilms suppress antitumor immunity, where M2 polarization of tumor‐associated macrophages (TAM), Treg recruitment, and CD8+ T cells are involved, and *F. nucleatum*–induced biofilm and NF‐κB signaling in M2 macrophages and a hostile TME [91]. *H. pylori* plays a role in programmed death‐ligand 1 (PD‐L1) and subsequent immune escape; thus, infection with this bacterium confirms the relevance of immune checkpoint upregulation [92]. Biofilm‐developing microorganisms, such as *F. nucleatum*, lead to chemoresistance against oxaliplatin and survive via favorable survival and inhibitory pathways [93]. Terpenoids diminish immunosuppression and can act as chemosensitizers [91].

However, it is important to note that the antibiofilm properties of terpenoids are not a general property of all microbial biofilms. Beneficial probiotic biofilms, like those of *Lactobacillus* spp. and *Akkermansia muciniphila*, enhance epithelial barrier function, immune homeostasis, colonization resistance, and the secretion of health‐promoting metabolites such as short‐chain fatty acids [94, 95]. Pathogenic tumor‐associated biofilms, however, formed by *F. nucleatum*, *H. pylori*, and enterotoxigenic *B. fragilis,* lead to chronic inflammation, immune evasion, and activation of oncogenic signaling pathways [96, 97]. There is evidence indicating that many dietary terpenoids tend to selectively inhibit quorum sensing, virulence factors, and overproduction of EPS, without broad‐spectrum depletion of the microbiota. This implies that terpenoids could be used specifically to inhibit the growth of pathogenic biofilms while allowing or even promoting beneficial microbiome functions, such as when used as dietary ingredients or directed nanoformulations [43, 98].

In vivo efficacy of terpenoids in combating biofilms is a limited approach but emerging. Thymol was found to significantly decrease the biofilm biomass, the production of extracellular polysaccharides, and the level of bacteria and inflammatory tissue damage, and to improve bacterial clearance in a mouse model of methicillin‐resistant *Staphylococcus aureus* (MRSA) biofilm infection [99]. Similarly, animal models of chronic bacterial infection showed carvacrol antibiofilm activity, decreasing EPS, colonization by microorganisms, and increasing susceptibility to antibiotics [100, 101]. Carvacrol potentially downregulates *icaA*, *icaB*, *icaC*, *agrA*, and *sarA* gene expression [101]. Even though these studies have focused on disrupting infectious biofilms rather than tumor‐associated biofilms, they do offer proof‐of‐concept that terpenoid disruption of EPS and inhibition of quorum sensing is effective under physiologically relevant *in vivo* conditions. Future studies using colorectal and GC models with polymicrobial biofilms need to be performed to further validate the findings in a translational context of biofilm‐associated carcinogenesis. However, more in vivo or preclinical research is needed to understand the long‐term impacts of terpenoids on the ecology of commensal biofilms and the host's internal microbial balance.

In CRC models, combinations of terpenoids with chemotherapeutics improved therapeutic and apoptotic responses and reduced metastasis. Preclinical evidence suggests these enhancements occur especially where biofilm‐mediated signaling creates resistance [91, 102].

However, the diverse terpenoids have potential strengths and limitations in the downregulation of the biofilm axis. The comparative analysis of diverse classes of terpenoids suggests that monoterpenoids show fast membrane‐active antibiofilm activity [103, 104, 105], while triterpenoids better modulate chronic inflammation and cancer signaling pathways related to tumor development (Table 3) [106, 107]. Sesquiterpenoids such as β‐elemene may be effective chemosensitizers for resistant tumor models (Table 3) [108, 109]. These patterns suggest that the terpenoid structural class may preferentially target bacterial biofilm, host inflammatory signaling, and tumor metabolic adaptation. This also provides a basis for the refinement of therapeutic structures.

**Table 3:** Comparative translational analysis of dietary terpenoids in biofilm‐associated cancer models.

Terpenoid	Structural class	Representative experimental model	Antibiofilm mechanism	Translational strength	Potential limitation	References
Thymol	Monoterpenoid phenol	*F. nucleatum* and *H. pylori* biofilm models (in vitro); prostate and colon cancer cell lines	QS inhibition, EPS reduction	Strong dual antibiofilm–anticancer activity	Rapid metabolism and volatility	[110, 111]
d‐Limonene	Monoterpenoid	Breast and lung cancer cell line models	Destabilization of biofilm matrix through EPS production	High dietary applicability	Poor aqueous solubility	[103, 104, 105]
Thymoquinone	Monoterpenoid quinone	CRC and breast cancer cell line models	Suppression of biofilm maturation, disrupting QS	Strong signaling pathway modulation	Low bioavailability and instability	[112, 113]
Geraniol	Monoterpenoid alcohol	Breast and prostate cancer models	EPS membrane destabilization	Effective microbiome modulation potential	Irritation and formulation instability	[77, 114, 115]
β‐Elemene	Sesquiterpenoid	Colorectal cancer models	Suppresses QS signaling and maturation of biofilms	Strong synergism with chemotherapy	Limited stability and delivery efficiency	[108, 109]
Triptolide	Diterpenoid	Pancreatic and breast cancer organoid/xenograft models	Disrupting the structural integrity of the biofilm and reducing the production of the EPS matrix	Highly potent anticancer efficacy	Severe systemic toxicity	[116, 117]
Ursolic acid	Pentacyclic triterpenoid	Gastrointestinal and pancreatic cancer models	EPS destabilization and disruption of cell membrane integrity	Strong anti‐inflammatory and antibiofilm effects	Poor water solubility	[106, 107]
Crocetin	Diterpenoid carotenoid derivative	Gastric and cervical cancer models	Disrupting QS	Strong antioxidant and apoptotic activity	Low intestinal absorption	[118, 119, 120]
Lupeol	Pentacyclic triterpenoid	Breast cancer models	Downregulation of virulence genes	Potential chemosensitizer	Limited clinical validation	[121, 122, 123]

## 4 Categories of Terpenoids in Cancer Prevention: Insight Into Terpenoid Type‐Specific Molecular Mechanism and Signaling Cascade

### 4.1 Monoterpenoid

#### 4.1.1 Thymol

**Figure 4:**
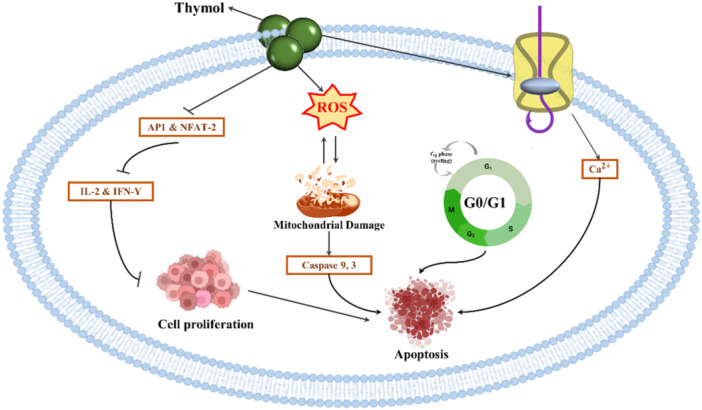
The molecular mechanism of thymol against cancer cells.

Monoterpene phenol derivative thymol has established profound anticancer activity with multiple mechanisms of action, hence its potential role as an anticancer drug. Thymol induces apoptosis, cytotoxicity, and cell cycle arrest as a potential anticancer mechanism (Figure 4) [124]. It affects PI3K/AKT/mTOR and MAPK signaling in metastasis and proliferation (Figure 4). The capability of thymol to interfere with several factors affecting cancer growth highlights its therapeutic potential. Oxidative stress induced by thymol interferes with mitochondrial function and subsequently triggers death in cancer cells. Studies have shown that thymol induces intrinsic and extrinsic cell death pathway activation via apoptosis. Thymol suppresses the viability and enhances the toxicity of prostate cancer cells in PC‐3 cells more than DU‐145 cells. These properties are often linked to the elevation of ROS, which triggers oxidative stress and prevents the mitochondria from functioning properly until apoptosis. Thymol has been validated to possess anticancer properties through an in vivo study [125]. Animal studies indicated that thymol caused the inhibition of tumor growth and cell proliferation. Thymol was found to demonstrate the suppression of tumor growth and the induction of apoptosis in mice injected with cervical and tongue squamous cell carcinoma cells. Thymol was identified to demonstrate antimetastatic activity in the lung metastasis model, which could be due to the inhibition of the WNT/β‐catenin pathway. These results confirm the capacity of thymol to suppress the process of cancer metastasis. The clinical results confirm the lower toxicity of thymol to mammals, which indicates its potential as an anticancer agent. Thymol is less effective than carvacrol in vitro. Thymol's specific mechanism of action against tumor cells causes death and a decrease in the viability of HepG2 cells [126]. Thymol acetic acid ester was found to be cytotoxic and genotoxic to colon cancer cells. Thymol docking assays reveal the strong binding affinity to protein molecules like PARP‐1 and protein kinase C, which confirms the role of thymol in cancer prevention and therapy [111].

#### 4.1.2 d‐Limonene

**Figure 5:**
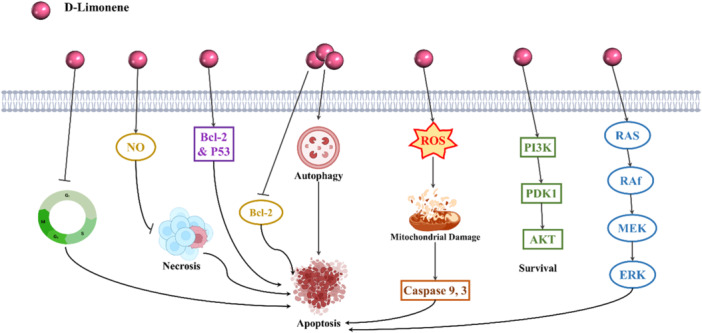
The molecular progression of d‐limonene against cancer cells.

d‐Limonene, a natural compound mainly found in citrus fruits, has drawn attention for its possible application in cancer treatment (Figure 5). At present, various mechanisms are known to be involved, mainly d‐limonene's actions on lung and breast cancers. Such apoptosis and DNA proliferation inhibition place d‐limonene in good standing for complementing cancer therapies. Apoptosis induction is the principal mechanism of the anticancer activity of d‐limonene (Figure 5). A study found d‐limonene to inhibit lung cancer cells from proliferation and reverse tumor growth in animal models through the promotion of autophagy and apoptosis [105]. This resulted in the induction of the expression of apoptosis‐related genes, indicating that d‐limonene triggers cellular mechanisms that cause programmed cell death. It was further observed that when autophagy was inhibited by chloroquine, d‐limonene‐mediated apoptosis was significantly inhibited. This majorly highlights that these two processes are interlinked in the anticancer activity mechanism of d‐limonene. Case studies on breast cancer have shown that d‐limonene results in the suppression of MCF‐7 cells, a commonly used line of breast cancer. Studies show that d‐limonene induced G2/M phase arrest through downregulation of key protein regulators Cyclin B1 and CDK1; thus, interfering with cell cycle progression causes raised apoptosis rates among treated cells (Figure 5) [127]. In support of this evidence of protein interaction with d‐limonene, computer‐aided assays provided further evidence of the molecularly targetable proteins of d‐limonene in mediating cell‐level mechanisms influencing cancer development. Clinical trials are now investigating the synergistic effect of d‐limonene in regular cancer therapies. Currently, d‐limonene in gel capsule form has been investigated in the prevention of xerostomia in patients with head and neck squamous cell carcinoma treated with radiation and platinum‐based chemotherapy. A mixture of d‐limonene and various anticancer drugs has been shown to produce synergistic effects [128]. Recently, a research study showed that a combination therapy involving d‐limonene and metformin, a well‐known antidiabetic agent that also has anticancer properties, significantly increased apoptosis rates in liver and breast cancer cells. This was because the combination led to alterations in the expression levels of pro‐apoptotic and antiapoptotic genes; hence, improving an anticancer treatment regimen [129].

#### 4.1.3 Thymoquinone

**Figure 6:**
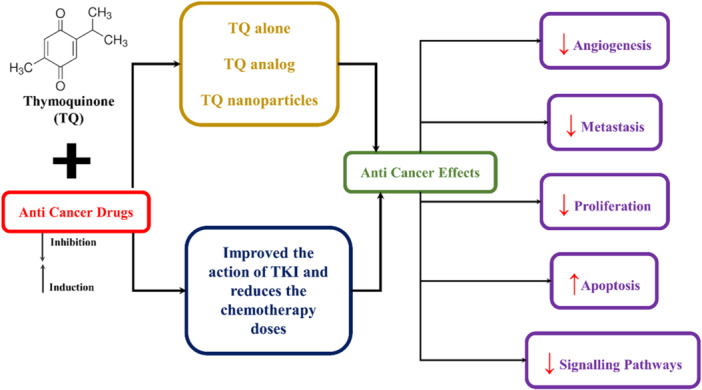
Thymoquinone on anticancer efficacy.

Thymoquinone (TQ) has been recognized for its various pharmacological properties, specifically its anticancer properties. TQ has gained traction within the area of oncology due to its potential to induce apoptosis in cancer cells, inhibit tumor growth, and modulate diverse signaling pathways (Figure 6). Its ability as a healing agent is being explored in both preclinical and clinical settings, with a focus on enhancing the efficacy of conventional cancer treatments [130]. The anticancer consequences of thymoquinone are attributed to several mechanisms (Figure 6). Apoptosis is induced via Bcl‐2‐family‐mediated changes to THQ, producing elevated ROS from a dysfunctional mitochondrion. In addition, THQ inhibits important survival signaling pathways like PI3K/Akt or NF‐κB in the promotion of inflammation and metastasis be multifaceted therapeutic agent to cause death of cellular function due to loss of cellular function (Figure 6). One of the more promising clinical applications of TQ is as a complementary therapy with existing therapies like chemotherapy and radiation therapy (RT). Studies to date have suggested that TQ causes significant sensitization of most cancer cells to chemotherapeutic agents, making them much more effective, all while reducing side effects. For example, research‐based evidence supports that TQ, when combined with doxorubicin, results in a significantly increased rate of apoptosis in doxorubicin‐resistant cancer cells, suggesting that TQ may play an important role in overcoming drug resistance and improving overall chemotherapy effectiveness [131]. Throughout the literature, there have been many reports of TQ having a synergistic effect with a variety of other chemotherapeutic agents for multiple cancer types. In a CRC study, TQ exhibited a significant increase in the efficacy of 5‐fluorouracil (5‐FU) compared to either agent alone, resulting in a significant decrease in tumor size. The ability of thymoquinone (TQ) to inhibit tumor growth while simultaneously inducing apoptosis allows for it to be utilized in combination regimens that may lead to stronger treatment modalities due to synergy. While there is a body of literature demonstrating TQ's ability to inhibit metastasis of tumors by means of down‐regulating the activity of matrix metalloproteinases (MMPs), which are involved in tumor invasion and extracellular matrix degradation [132]. Animal and human studies suggest that TQ can inhibit cell migration by modulating the focal adhesion kinase and ERK phosphorylation pathways in breast cancer and glioblastoma models. Inhibition of metastasis is beneficial for improving patient outcomes, as well as preventing the spread of cancer. In addition to its direct anticancer effects, TQ is recognized for its immunomodulatory effects, which may enhance the immune response to tumors.

Thymoquinone may potentially restore immune function to the antitumor immune response in the TME by modulating immune cell activity and inhibiting the NF‐κB signaling pathway. In so doing, thymoquinone may enhance the effectiveness of immunotherapeutic agents by resulting in a more effective antitumor immune response; thus, thymoquinone would be an appealing candidate for use in combination with immunotherapy [30]. There have been numerous preclinical trials demonstrating the anticancer activity of thymoquinone in a range of cancers, including colon, ovarian, breast, and lung malignancies. These studies have continuously reported a substantial reduction in tumor size and increased survival rates when thymoquinone is administered alone as well as when given in combination with other anticancer therapies. While the results from these studies are encouraging and indicate substantial therapeutic value, additional clinical trials assessing the dose‐response and safety profile of thymoquinone will be required to translate these results to clinical practice. Moreover, while an abundance of preclinical evidence exists to support thymoquinone's anticancer effects, few clinical trials have been conducted to evaluate the therapeutic effect of thymoquinone in humans [133]. Some early‐phase clinical trials are currently underway to establish the safety and efficacy of thymoquinone for the treatment of certain types of cancer. The goal of these studies is to establish optimal dosing regimens and to assess how well thymoquinone will combine with currently used anticancer therapies. Thymoquinone is generally viewed as a compound that has an acceptable level of risk associated with it (i.e., low toxicity). When thymoquinone is ingested or given to patients via injection at therapeutic dosages, it creates a higher concentration of the compound in the body, inducing apoptosis (programmed cell death) in most cancer cells while leaving normal (healthy) cells virtually unaffected. Although thymoquinone is not generally known to cause any unwanted effects, like many chemotherapies [134]. However, long‐term safety studies regarding thymoquinone and its ability to interfere with medication utilized for conditions that cancer patients face need to be completed as well.

#### 4.1.4 Auraptene

**Figure 7:**
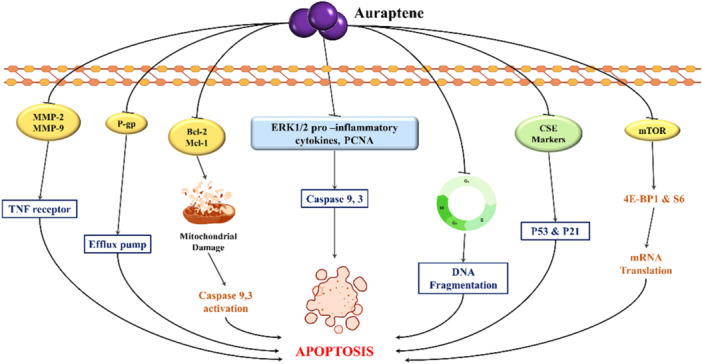
The molecular mechanism of auraptene‐induced cancer cell apoptosis.

An herbal bioactive compound called auraptene falls under the classification of monoterpene coumarin ether, mainly found in a variety of citrus fruits and some other plant materials belonging to the Rutaceae. It was first isolated in the early 1930s, and it has subsequently been identified for several pharmacological properties. Auraptene belongs to the prenyloxycoumarin magnificence and is known for its distinctive chemical structure, which contributes to its biological activities. Its molecular components are C_19_H_22_O_3_, and it is characterized by the presence of a geranyloxy group attached to the coumarin backbone [135]. The therapeutic ability of auraptene has been significantly studied, revealing a wide range of useful outcomes. One of its most extraordinary properties is its chemopreventive activity toward numerous forms of cancer, including liver, skin, tongue, esophagus, and colon cancers. In preclinical research, the use of rodent models of auraptene has verified full‐scale inhibitory results on tumorigenesis and cancer cell proliferation. The mechanisms underlying these anticancer effects involve modulation of key intracellular signaling pathways that modify cell proliferation, inflammation, and apoptosis (Figure 7). Auraptene's anticancer properties are attributed to its potential to induce apoptosis in most cancer cells while sparing normal cells (Figure 7) [136]. It has been proven to activate phase II detoxifying enzymes together with glutathione S‐transferase (GST) and quinone reductase (QR), which play important roles in detoxifying cancer‐causing agents and defending cells from oxidative stress [137]. Additionally, auraptene has been discovered to inhibit MMPs, which are involved in most cancers' cellular invasion and metastasis [138]. By focusing on these pathways, auraptene efficiently reduces tumor growth and forestalls the progression of cancer. In addition to its anticancer effects, auraptene reveals strong anti‐inflammatory and antioxidant properties [139].

This double activity means that auraptene could well be a therapeutic compound for inflammatory diseases, such as arthritis and colitis, as well as for chemoprevention. Auraptene's neuroprotective properties have additionally garnered attention in current studies. Studies suggest that it can enhance neuronal survival by means of stimulating the production of neurotrophic elements such as glial cell‐derived neurotrophic factor and brain‐derived neurotrophic factor. These components are important for the growth, differentiation, and preservation of neurons and suggest an application for neurodegenerative disorders such as Parkinson's disease. Another feature of auraptene, which enhances its attractiveness as a therapeutic agent, is the protection profile. Auraptene's safety profile is a desirable attribute for inclusion in dietary supplements or targeted foods for cancer prevention and healthy living marketing [140]. Auraptene's pharmacologic activities go beyond the prevention of cancer, and it also has antibacterial, antifungal, and antidiabetic properties. It has been proven that auraptene can inhibit the growth of various pathogenic microorganisms and fungi, which demonstrates the potential for use as an antimicrobial agent in herbs. Furthermore, its capacity to modulate glucose metabolism indicates that it is able to be useful in dealing with diabetes‐associated headaches. Although the results of these therapeutic programmes with auraptene are encouraging, more human clinical studies are necessary to definitively understand its therapeutic effects and how it works. The majority of the information in today's world is acquired from preclinical models, and the findings in the preclinical models will have to be evaluated in well‐designed scientific trials in order to test them [141].

#### 4.1.5 Eugenol

A specific aromatic compound, which is classified more generally as a phenylpropanoid, is found in essential oils from many flowers, including clove (*Syzygium aromaticum*), nutmeg, cinnamon, and basil. It is a compound with the chemical formula C_10_H_12_O_2_ and has a special, high‐spiced, clove‐like smell [142]. Eugenol is a pale yellow to colorless oily liquid that possesses several medicinal properties, making it treasured in both traditional and cutting‐edge medicinal drugs. The molecular structure of eugenol consists of a phenolic ring with an allyl side chain and a methoxy group, which was isolated as 4‐allyl‐2‐methoxyphenol. The structural configuration helps to contribute to organic activity and balance. Eugenol is soluble in natural solvents like alcohol, ether, and chloroform, but is only partially soluble in water. Its melting point is approximately −7.5°C, at the same time as its boiling point is around 254°C, indicating that it exists as a liquid at room temperature [143]. Eugenol shows remarkable antimicrobial potential against a variety of pathogens such as bacteria, fungi, and viruses. Eugenol has an effective anti‐inflammatory and antioxidant effect as well as an antimicrobial effect [144]. Studies show that eugenol has demonstrated anti‐inflammatory effects on the production of pro‐inflammatory cytokines and on the inhibition of markers of oxidative stress in various experimental models. This will help eugenol to be a potential compound to prevent chronic diseases associated with oxidative damage and also aid in its healing properties in inflammatory conditions. Eugenol's anticancer properties have additionally been investigated. This has been demonstrated to induce programmed cell death (apoptosis) of cancer cells, while leaving normal cells intact. It does this through the modulation of important signal transduction pathways that regulate cell survival and proliferation, such as by inhibition of NF‐κB signaling and activation of caspase cascades. Furthermore, eugenol has been shown to halt the proliferation of various types of cancer, such as breast, colon, and prostate cancer, in a variety of cancer models [145]. While eugenol is commonly considered safe to use in meals and medicinal products at recommended doses, it could cause slight side results inclusive of pores and skin irritation or hypersensitivity reactions, in some individuals. Long‐term exposure or consumption of large amounts could also have more serious negative effects, such as liver damage or seizures [146]. So, it is crucial to follow the recommended guidelines for its consumption for protection purposes. However, encapsulation of eugenol has been investigated as a strategy to make it look good and make it bioavailable. To enhance the transportation of eugenol in healing applications, techniques combined with stable lipid nanoparticles/microemulsions can be useful because they will protect the eugenol from rapid metabolism and degradation. The improvements can also help to enable more effective applications of eugenol in the field of medicine.

#### 4.1.6 Geraniol

**Figure 8:**
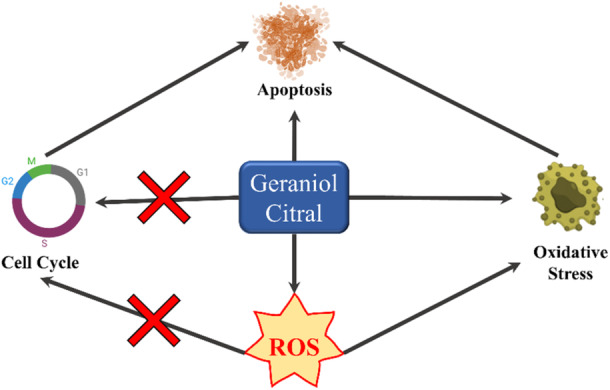
A general mechanism of action of the anticancer effects of geraniol.

Geraniol is an obvious going on monoterpenoid alcohol widely known for its therapeutic properties. With the chemical components C_10_H_18_O, geraniol is characterized by its satisfactory rose‐like scent, making it a key factor in lots of vital oils, along with rose oil, citronella oil, and palmarosa oil. It is an acyclic compound with a structure that incorporates a hydroxyl group (–OH) connected to an extended carbon chain, specifically classified as 3,7‐dimethylocta‐2,6‐dien‐1‐ol [147]. Geraniol is a compound with a broad spectrum of biological activities, which has attracted enormous interest in pharmacology and natural product chemistry. One of its most amazing properties is its antimicrobial activity. Geraniol has been clinically demonstrated to have antibacterial, antifungal, and antiviral activity and is effective against a wide range of pathogens [148]. It has, for example, been shown to be effective against gram‐positive and gram‐negative microorganisms and fungi like *Candida albicans*. This antimicrobial activity helps its use in food preservation and as a natural alternative to synthetic preservatives. Apart from its antimicrobial activity, geraniol was also studied for its anti‐inflammatory and antioxidant properties (Figure 8). It has been shown to have anti‐inflammatory effects by blocking the production of pro‐inflammatory cytokines, and its effect in reducing the markers of oxidative stress has been demonstrated in various experimental designs (Figure 8). Geraniol is a candidate for the control of inflammatory diseases such as arthritis and various chronic diseases due to oxidative damage, thanks to its dual action. In recent years, the anticancer properties of geraniol have also been of interest. Studies imply that it could result in apoptosis (programmed mobile loss of life) in most cancers cells at the same time as sparing normal cells. This effect is mediated through the regulation of important signaling pathways of cell survival and growth. For example, geraniol is reported to suppress the NF‐κB signaling pathway and has been found to suppress the growth of various forms of cancer in different cancer models, including breast cancer and prostate cancer [114, 149]. The metabolism of geraniol within the human body is, in general, the process of enzymatic conversion in the skin cells by cytochrome P450 enzymes. The results reveal that the investigated enzymes are crucial in the biotransformation of geraniol to different metabolites. It is essential to know the metabolic pathway to evaluate the safety and efficacy of geraniol‐containing products in therapeutic use. Although geraniol has several benefits, there are concerns about its safety. Usually safe to use at proper concentrations, but excessive exposure can lead to infection of the skin or allergy in some individuals. Geraniol has been listed as a possible irritant by regulatory agencies, and therefore, care must be taken when formulating products that contain the compound [50]. Moreover, the developing demand for natural components has led to an increased interest in geraniol's utility in aromatherapy and natural health products. Recent improvements in biotechnology have opened new avenues for the sustainable production of geraniol. Researchers are exploring genetic engineering techniques to improve the biosynthesis of geraniol, including the use of microorganisms including *E. coli* and *S. cerevisiae* [148]. These strategies' goal is to optimize production approaches and decrease reliance on traditional plant extraction techniques.

### 4.2 Sesquiterpenoids and Sesquiterpene Lactones

#### 4.2.1 Ambrosin

Recent studies have begun to explore the role of ambrosin—a sesquiterpene lactone derived from the South American medicinal plant *Ambrosia arborescens*—in the treatment of cancer. The compound has shown considerable anticancer activity, particularly against drug‐resistant types of breast cancer cell lines. These properties, namely, the induction of programmed cell death, along with the inhibition of cell growth, make ambrosin an excellent potential candidate for future drug development for use in oncological therapy. Research showed the effects of ambrosin on MDA‐MB‐231 cells, a well‐accepted model for triple‐negative breast cancer, renowned for being aggressive with flat, spread cells that produce multiple copies of certain drug resistance genes. Assays, including WST‐1 for cell viability and flow cytometry for assessment of apoptosis, were used [150]. The results showed that ambrosin significantly decreases cell viability at an IC_50_ of 25 µM and exerts potent cytotoxicity against the cancer cells while exhibiting minimal toxicity against normal breast cells (MCF‐12A). This selectivity is crucial for developing effective cancer treatments with fewer side effects. Ambrosin induces apoptosis, which is its main mechanism of action against cancer [150].

Further, ambrosin treatment resulted in dramatic morphological changes in MDA‐MB‐231 cells, including increased membrane blebbing, which is characteristic of apoptosis [151]. An analysis of flow cytometry revealed a marked increase in cells that were considered to be apoptotic between control and high concentrations of ambrosin (3.5% to approximately 56%). This apoptotic effect was found to be correlated with changes in the expression of some apoptosis‐related proteins, specifically an increase in pro‐apoptotic Bax and a decrease in antiapoptotic Bc1‐2, showcasing ambrosin's role in promoting apoptosis through activation of caspases. Ambrosin itself is a known producer of oxidative stress in cancer cells through enhanced production of reactive oxygen species. Ambrosin induces a rise in oxidative stress in neoplastic cells through the elevation of ROS levels. It is well known that oxidative stress initiates apoptosis and may cause mitochondrial impairment, promoting the demise of cancerous cells [152]. This study also assessed the effects of ambrosin on the Wnt/β‐catenin signaling pathway frequently disrupted in tumors. Ambrosin inhibited the activity of this pathway in a dose‐dependent manner. This inhibition may be significant in reducing tumor growth and metastasis by restoring normal cellular communication. In addition to these direct antineoplastic actions, the potential for combination strategies with ambrosin is being explored. Studies showed that a mixture of ambrosin and regular chemotherapy may improve overall survival against aggressive breast tumors, thus reducing resistance mechanisms regularly induced in their course [153].

#### 4.2.2 Artesunate and Artemisinin

Artesunate and artemisinin, both compounds, have recently been taken into consideration for therapeutic studies for their cancer treatment potential. Most of the research conducted on these drugs has focused on uncovering mechanisms and other properties of these drugs as anticancer agents in different cancers [154, 155]. A clinical trial conducted in 12 patients with relapsing gliomas has been performed with artesunate at 200 mg a day, along with ongoing treatment with temozolomide. The results indicated good tolerance of artesunate, including only one case of transient hematological toxicity. A small series reported encouraging median survival of artesunate combined with temozolomide: 5 months in those with late‐stage disease at diagnosis, 46 months in those who were kept in the remission stage. The study also included some evidence suggesting that artesunate may act as both a cytotoxic agent against glioblastoma cells and a supportive therapy that enhances the efficacy of already existing treatment options like temozolomide [156]. In general, the study mentions modulation of intracellular signaling pathways generating oxidative stress and inhibition of angiogenesis, as processes necessary for tumor growth and metastasis. The study's emphasis was more about the point that artemisinin, capable of overcoming drug resistance, may be very useful against cancers poorly responding to conventional therapies [157]. In addition to glioblastoma, several clinical trials have investigated the use of artesunate in treating CRC. In this randomized study, which always consisted of 30 patients in each arm, and included 2 weeks before surgery, patients receiving artesunate (at 200 mg/day) had more tumor cells undergoing apoptosis than placebo‐treated patients. Findings indicate that such artesunate could increase the apoptotic processes in malignancy, which may help improve surgical outcomes and decrease rates of recurrence. In addition, recent studies have pointed to artesunate‐ferritin interactions in cancer cells [158]. High levels of ferritin are often correlated significantly with poor prognoses for cancer patients because of their role in supporting tumor growth. Artesunate can, however, lower the levels of ferritin in cancer cells, making them more susceptible to other treatments. This mechanism of action complements the ability of the drug to kill iron‐rich environments typical of rapidly dividing cancer cells.

#### 4.2.3 β‐Elemene

**Figure 9:**
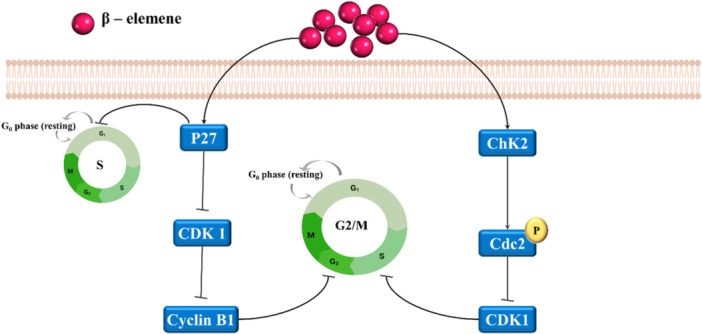
The molecular processes via which β‐elemene affects cancer cells.

Recent clinical studies have highlighted the potential of β‐elemene as a promising cancer treatment, thereby highlighting its antitumor effects upon certain cancers such as lung, breast, and liver cancers. Its mechanism of action includes induction of apoptosis, inhibition of cell proliferation, and multiplication of the effectiveness of conventional chemotherapy drugs. The efficiencies of β‐elemene and chemotherapy in patients with advanced non‐small cell lung cancer cells (NSCLC) were compared in a clinical trial [159]. A total of 35 clinical trials have been conducted among more than 2700 patients and showed that β‐elemene significantly increased the response rate and disease control rate in patients undergoing chemotherapy, especially when given for fewer than 6 cycles [159]. The synergistic effect of β‐elemene on tyrosine kinase inhibitor (TKI)‐resistant NSCLC has been proven to be due to its apoptosis‐inducing action triggered by the AMPK and MAPK signaling pathways (Figure 9) [160]. The study indicated that β‐elemene could be a valuable therapeutic option for patients with TKI‐resistant tumors, potentially overcoming resistance mechanisms. There has also been an examination of the role of β‐elemene in the treatment of acute myeloid leukemia (AML). In a trial‐based data set from 120 patients, refractory/relapsed AML patients who received β‐elemene in combination with traditional treatment regimens achieved an overall effective rate of 80.8% versus 52.9% in a control group. Moreover, β‐elemene materially augments an AML treatment's responsiveness, thus providing a new approach toward the amelioration of patient outcomes in this difficult disease [161]. Mechanistically, β‐elemene has been shown to exert its anticancer effects through multiple pathways. The cell cycle arrest in the phases G0, G1, G2, or M is achieved via the induction of apoptosis through modulation of the expression levels of pro‐apoptotic and antiapoptotic proteins, as well as the suppression of the EMT process for the prevention of angiogenesis and metastasis, and all these diverse functions make it a potent antitumor agent against different types of cancer [162].

#### 4.2.4 Triptolide

Triptolide has demonstrated significant anticancer properties within various types of cancer, which include pancreatic, breast, and lung cancers. Because of the problem of high levels of toxicity coupled with poor solubility, its efficacy for clinical use has received limited application. To address issues surrounding triptolide toxicity and solubility, several formulations and analogs have been discussed to deliver greater therapeutic efficacy with minimal side effects [163]. Of all recent advancements in triptolide research, the notable mention must be of Minnelide, a water‐soluble prodrug of triptolide. One study showed the efficacy of Minnelide in pancreatic cancer patients. The results found that the patients treated with Minnelide and normal chemotherapy patients had significantly better OS than the chemotherapy‐only patients. This study gave major supporting evidence for the use of Minnelide as an effective adjunct therapy in managing the disease with its bad prognosis and limited options for treatment [164]. In addition to Minnelide, a new triptolide analog called CK21 has been synthesized to improve its pharmacokinetics and reduce its toxicity. Another study assessed CK21 using pancreatic cancer preclinical models. Data from this study showed that CK21 significantly inhibits tumor growth via the NF‐κB pathway in xenograft models and patient‐derived tumor organoids with minor toxicity. This study confirms CK21 as an attractive candidate for clinical evaluation, aimed at providing a much safer choice compared to triptolide. Triptolide's effects on breast cancer have also been highlighted. A bibliometric study indicated that triptolide targets different miRNAs, which regulate cell proliferation, migration, and apoptosis in breast cancer models [165]. Preclinical studies indicated that triptolide has potential synergistic properties with existing treatment strategies, as it successfully overcomes resistance mechanisms of apoptosis that are often observed in aggressive breast cancer models. This could support and justify the adjunctive role of triptolide in the management of patients with breast cancer that is innately refractory to available treatments at that moment. Lastly, studies indicated that triptolide could play a pivotal role in reversing drug resistance mechanisms in non‐small cell lung cancer patients. A more recent study indicated that triptolide made NSCLC cells sensitive to available chemotherapeutic treatments by targeting different signaling pathways that play a crucial role in resistance mechanisms [166].

### 4.3 Diterpenoids

#### 4.3.1 Crocetin

Saffron (*Crocus sativus*) is supposed to be a good cancer treatment. There exists evidence that it presents anticancer activity against different types of cancers, including breast (MDA‐MB‐231 and MCF‐7 cell lines), gastric, and cervical cancers. The potential pathway includes induction of apoptosis and inhibition of cell proliferation [167]. The study also highlighted the fact that treatment with crocetin upregulates the pro‐apoptotic protein Bax while downregulating the antiapoptotic protein B‐cell lymphoma 2 (Bcl‐2) [167]. The drug impeded MMPs, or a group of enzymes, utilized by the cancer cells to metastasize through tissues. In another study against GC, it was observed that crocetin had a strong anticancer effect by inhibiting AGS gastric adenocarcinoma cells [168]. Further, crocetin has demonstrated a remarkable decrease in cell growth, with apoptosis marked by morphological changes and activation of caspase‐3. Other findings highlighted the importance of downregulation of the Bcl‐2/Bax ratio due to crocetin in favor of apoptosis. Crocetin enhances the action of a chemotherapeutic drug called fluorouracil, meaning its use as adjuvant therapy in relation to GC can bring good results. The use of crocetin has also been elucidated for cervical cancer. A study found the effect of crocetin on the inhibition of HeLa cells in vitro as well as its capacity for cell cycle arrest in the G1 phase [118]. These were attributed to the upregulation of p21WAF1/Cip1 and the downregulation of cyclin D1. In addition, the study found that crocetin also presented a synergistic effect with vincristine, leading to a significant increase in apoptotic effects in cervical cancer cells. Finally, the findings of this study suggest that crocetin could act as a potential agent that would increase the efficiency of the existing chemotherapeutic agent, averting the adverse effects of the existing treatments [169].

#### 4.3.2 Phytol

For the past few years, studies have concentrated on phytol, a natural compound obtained easily from certain parts of different plants, in searching for alternatives in the treatment of cancer. Phytol has displayed very high anticancer potential across cancers of different types, including breast, cervical, and leukemia. It has one major mechanism of action that includes apoptosis induction, inhibition of cell proliferation, and enhancement of chemotherapeutic agents in being efficient [170]; in vitro anticancer studies of phytol extracted from *Gracilaria edulis* were performed on human breast cancer cell lines. It was shown that phytol exhibited anticancer properties at IC_50_, estimated to be 125 µg/mL, thus increasing the possibility of treating breast cancer. The authors indicated that increasing concentrations of phytol dramatically reduced cell viability in a dose‐dependent manner. Phytol is effective against cervical cancer and inhibits cell proliferation, inducing apoptosis in HeLa cells [79]. The effect of phytol on leukemia cells has also been studied. It was demonstrated that phytol exerted cytotoxic activity against different leukemia cell lines, considering doxorubicin‐resistant sublines [171, 172]. Phytol induced the arrest of the cell cycle, mainly in G2/M, and caused apoptosis by activating the caspases [171, 172]. Research has further shown phytol to be capable of enhancing the effectiveness of conventional chemotherapeutics. Studies demonstrated phytol's synergistic effects with drugs like cisplatin and doxorubicin. Their combinatory therapeutic strategies enhanced anticancer efficacy, while overcoming drug resistance in a few cancers [173]. This suggests that adding phytol in cancer treatment regimens can potentially improve overall therapeutic benefit to patients who are dealing with resistant cancers.

#### 4.3.3 Ursolic Acid (UA)

**Figure 10:**
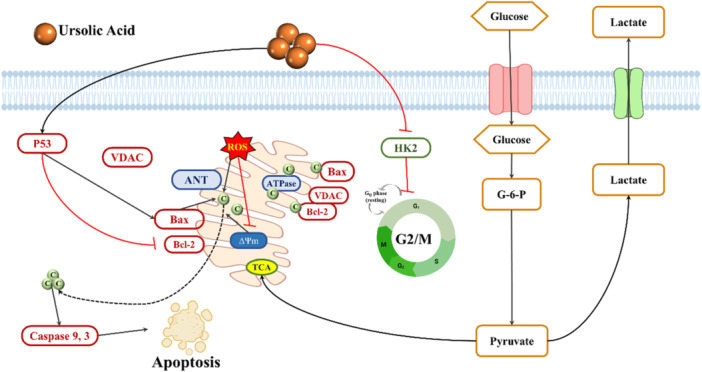
The molecular processes of ursolic acid in cancer cell inhibition.

In recent years, studies have focused on UA, a natural compound found in various fruits and herbs used in cancer treatment. Several research studies have demonstrated that UA has significant anticancer effects against breast, pancreatic, and gastrointestinal cancers [174]. UA can inhibit cell proliferation, induce apoptosis, inhibit the uncontrolled proliferation of these cells, and modulate the key signaling pathways involved in the initiation of tumors (Figure 10) [175]. This study, therefore, highlights the potential of UA as an effective agent against gastrointestinal cancers, suggesting effectiveness in combination with existing treatment methods. Besides inhibiting gastrointestinal cancers, UA has also been promising with pancreatic cancer, which is highly aggressive and is often resistant to standard therapies. Research also shows that UA can increase chemosensitivity against gemcitabine, a standard drug treatment for pancreatic cancer. Besides inducing apoptosis and autophagy, UA inhibited migration and invasion in pancreatic cancer cells through regulating potential signaling pathways such as NF‐κB and PI3K/Akt/mTOR (Figure 10). This suggests UA could supplement the treatment of clinical patients and offer better survival chances. The effects of UA on breast cancer cells have also received a considerable amount of research scrutiny. One such investigation found that UA induced an inhibition of viability and migration through various breast cancer cell lines by directly targeting multiple signaling pathways that include PI3K/Akt and NF‐κB [176, 177]. Moreover, UA was seen to trigger apoptosis and autophagy in these cells, leading to cell cycle arrest. The data given indicate UA has potential as a breast cancer treatment for further research exploring possible mechanisms of resistance in aggressive forms. Further, novel delivery methods to enhance UA's bioavailability have also been explored by researchers, as it has limited tissue penetration owing to its poor solubility. In fact, other recent studies have shown nanoparticles to deliver UA more efficiently to the tumor sites. For example, UA‐loaded liposomes, preclinically, display a distinctly improved anticancer efficacy and a favorable safety profile as compared to traditional formulations [106].

### 4.4 Triterpenoids

#### 4.4.1 Betulinic Acid (BA)

**Figure 11:**
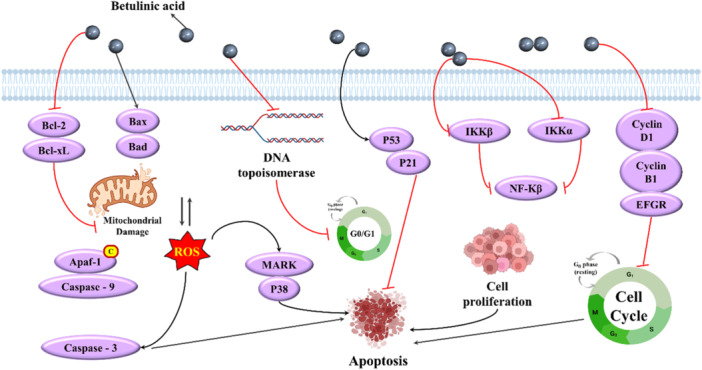
The anticancer molecular processes of betulinic acid.

BA was reported to have potent anticancer activity against different forms of cancer, having been effective against melanoma, breast cancer, glioblastoma, and leukemia. The mechanism of action primarily involves induction of apoptosis, inhibition of cell growth, and enhanced activity of conventional chemotherapy agents [178]. Research has demonstrated that BA significantly improved the effectiveness of Taxol (Paclitaxel) on breast cancer cells by inducing ER‐mediated apoptosis. The immunostaining results revealed that GRP78 was a target for BA and disrupted the PERK/GRP78 complex formation, which leads to an increase in apoptosis and arrest in the G2/M‐phase cell cycle due to disrupted PERK activity. The study revealed that GRP78 silencing and overexpression can increase and reduce, respectively, the protein level and activity of PERK in MCF‐7 cells [178]. These results suggest that betulinic acid may be a valuable breast cancer chemosensitizer to improve outcomes when used with current therapies. Betulinic acid has impressive effects against glioblastoma, aside from breast cancer. A study revealed that BA induced apoptosis and inhibited the proliferation of the T‐lymphoblastoid leukemia cell line (MOLT‐4) and DCPM‐4 resistant leukemia through mitochondrial pathways (Figure 11) [179]. Furthermore, BA has shown the greatest activity on primary leukemia samples that resisted standard chemotherapeutic agents and suggested a blockade of drug resistance, which in reality is greatly observed in leukemia treatment. In addition to this, new drug delivery systems have increased the bioavailability and therapeutic efficacy of BA. Studies have indicated that BA encapsulated in lipid‐based carriers greatly enhances its anticancer activity in preclinical models, when compared with free BA formulations [180]. Such an approach ensures not only a greater therapeutic outcome but also creates a foothold for an effective advancement in the clinical applications of betulinic acid.

#### 4.4.2 Lupeol

Lupeol belongs to the triterpene category and has exhibited anticancer potential against breast cancer, non‐small‐cell lung carcinoma, and melanoma [123]. The potential mechanisms include apoptosis induction, cell proliferation inhibition, and modulation of the most critical signaling pathways. In a study, lupeol efficacy against erlotinib‐resistant NSCLC cells that are resistant to erlotinib (H1975) has been observed [181]. Further, lupeol potentially decreases cell viability and induces apoptosis within these resistant cells. Lupeol has been shown to decrease cell viability and promote apoptosis within these resistant cells. Further, apoptosis is caused by lupeol through the downregulation of epidermal growth factor receptor (EGFR) and STAT3 pathways. More specifically, lupeol lessened the phosphorylation of its targets, which are EGFR and STAT3, and reduced the expression of STAT3 target genes, cyclin D1 and survivin [182]. Another study evaluated the anticancer activity of lupeol on A549 human lung adenocarcinoma cells [183, 184]. Lupeol caused an LD_50_ of about 62.53 mg/mL and also caused a noticeable dose‐dependent antiproliferative effect on the cells. The study utilized a variety of apoptosis and cell migration assays, revealing that lupeol induced apoptosis. Further supporting lupeol's role as an effective chemotherapeutic agent for NSCLC, lupeol downregulated genes such as Bcl‐2 and Bcl‐xL, among others, in treated cells. The effects of lupeol have also been such that models of breast cancer have been developed. The study explained that lupeol diminished breast cancer cell proliferation while enhancing apoptosis that modulated key signaling pathways engaged in the causes of cell life and death. According to research, the anticancer activity of lupeol might depend on disrupting most critical cellular processes, which favor the growth of tumors, and this has identified it as another suitable candidate for combination therapies with existing chemotherapeutic agents [185]. Lupeol has been shown in studies to reduce cancer cell migration and invasion by affecting the expression of proteins involved in the EMT. Based on that, lupeol could not only be an anticancer agent but could also operate as a metastasis‐preventing agent, which indeed remains an imminent platform as far as cancer treatment is concerned [121]. Lastly, studies have been conducted investigating some novel formulae to increase the bioavailability of lupeol. These researchers now propose nanoparticle‐based delivery systems focused on improving the solubility and bioavailability and targeting lupeol more directly to the tumor site. In so doing, systems are now particularly designed to ameliorate therapeutic potential with the least side effects [186].

## 5 Food, Gut Microbiome, and Terpenoids in Biofilm‐Mediated Cancer Mitigation

Dietary factors are key modulators of the gut microbiota, and novel evidence supports a role for functional foods in facilitating beneficial microbial alterations with carcinogenic potential, mainly in colorectal and GCs. Various food compounds, including plant bioactives, fibers, and fermented foods, modulate gut microbiota and its metabolism by increasing phylum levels of beneficial microbes like *Bifidobacterium* and *Lactobacillus*, with a consequent increase in short‐chain fatty acids, hence strengthening the mucosal barrier and anti‐inflammatory conditions [53]. The gut microbiota acts as a biochemical–immunological interface where bioactive food compounds and phytochemicals are metabolized and activated to modulate host inflammation, immune, and signal transduction in carcinogenesis [187]. Yogurt, a dietary component with natural concentrations of probiotics, has been shown to reduce the incidence of specific subsets of CRCs by modulating bacterial gut microflora and minimizing conditions of chronic inflammation [188]. Functionally fortified foods with terpenoid active extracts, as in citrus limonene in beverages and thymol/ursolic in plant baselines, could act in a bacterial microbiota interface mode to modulate biofilm formation in *F. nucleatum* and *H. pylori* discharged tumor microzones by impeding NFk/STAT3 signaling for immune reinstatement [187].

Further, functional foods encapsulated with nanotechnology could enhance terpenoids' bioactive stability, bioavailability, and targeted delivery in food formats that are ingestible. The terpenoids' lipophilicity is solubilized using nano‐emulsions, nanolipids, and biopolymer nanocarriers, which protect against stomach enzymatic degradation and control release in the gastrointestinal tract and its pathologies [38, 189]. In bioactive food and biological applications, nano‐emulsion beverages, nano‐prepared food‐delivery films, and biopolymer nanocarriers encapsulating plant extracts have presented an amplified antibacterial and anti‐inflammatory response in controlled biological and food assays [190]. Generally recognized as safe (GRAS)‐compatibility and targeting bioactive interfaces in colorectal and GC pathology and microbiology using nanotechnology‐mediated biological delivery are feasible and relevant in cancer treatment and prevention strategies. Biofilms derived from microorganisms belonging to *Fusobacterium* and *Helicobacter*, involved in bioactive food intervention strategies and carcinogenetic pathways, could be modulated using terpenoids encapsulated in biopolymer nanocarriers and targeting pathways involving NF‐κB/STAT3 [191, 192, 193].

## 6 Nano‐Mediated Target Delivery of Terpenoids in Cancer Treatment: Translational Integration of the Antibiofilm–Anticancer Axis

While dietary terpenoids have antibacterial and anticancer properties, their translational potential is limited due to aqueous solubility, gastrointestinal instability, metabolism, and poor bioavailability at tumor sites (Table 3). Further, these pharmacokinetic constraints also limit their ability to effectively penetrate polymicrobial biofilms and microbial niches found in tumors [194]. It is thus important to consider the use of nano‐enabled food systems for delivery rather than just passive delivery vehicles, as they are active delivery platforms that might be used to increase the accumulation of terpenoids in the TME that is rich in biofilms. It creates a mechanistic connection between the concept of microbial dysbiosis, biofilm‐related carcinogenesis, modulation of terpenoid signaling, and functional nanonutrition. Nanoformulations thus appear as technologies that make possible the implementation of the proposed dietary antibiofilm–anticancer axis.

Moreover, the therapeutic efficacy of nano‐formulated terpenoids against biofilm‐associated cancers is not only attributed to improved solubility, but also due to the ability of the nano‐formulations to invade the EPS, the major physicochemical barrier of microbial biofilms [195]. EPS act as a steric barrier, an electrostatic barrier, and a hydrophobic barrier and limit the diffusion of nanoparticles. Size, surface charge, hydrophobicity, and surface functionalization primarily determine the penetration of nanoparticles [196]. Particles smaller than 100 nm tend to diffuse more easily through the water channels in the biofilm, while larger particles are absorbed into the polysaccharide matrix. Neutral or slightly anionic nanoparticles are shown to penetrate well and prevent electrostatic interactions with acidic polysaccharides and eDNA, while controlled cationic modification allows for better retention within biofilms [197]. Local EPS degradation using enzyme‐functionalized nanocarriers (EPS degraders) containing DNase I, dispersin B, or proteases results in deeper diffusion and controlled release of terpenoids, while surface engineering with polyethylene glycol reduces the nonspecific adhesion to EPS [198]. In addition, nanocarriers with biofilm responsiveness take advantage of the acidic pH, bacterial degradants, and increased levels of ROS for targeted drug delivery at the site of the biofilm [199, 200]. The improved penetration of EPS through nanoparticles results in the disruption of quorum sensing, disruption of biofilm architecture, enhanced immune‐cell accessibility, and suppression of biofilm‐associated NF‐κB, STAT3, and Wnt/β‐catenin signaling, which helps to boost the antibiofilm and anticancer efficacy of dietary terpenoids [201].

**Figure 12:**
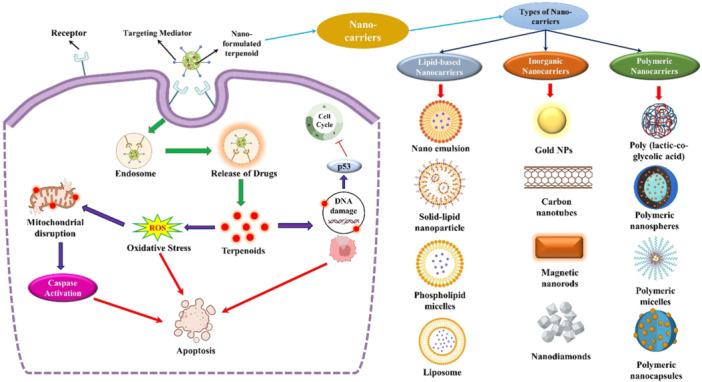
Cancer prevention mechanisms of nano‐formulated terpenoid substances.

Furthermore, nanoformulations increase the safety of terpenoids by improving their reduced cytotoxicity and overall intake of the drug in the body through targeted distribution for specific tissues or cells (Table 4) [202]. The anticancer mechanisms of nanoformulated terpenoids entail multiple processes, including the inhibition of tumor proliferative activity, apoptosis, DNA damage, or mitochondrial damage (Figure 12) [203].

**Table 4:** NPs mediated precision delivery of terpenoids for biofilm‐mediated tumor environment.

Delivery method	Name of the terpenoid involved	Nanocarrier	Advantage	Cancer causes biofilm targeting significance	Reference
Nanoemulsions	Thymol, UA, limonene	Nanoemulsions of oil‐in‐water or emulgels	Upsurge in the solubility of terpenoids Improved mucosal adhesion and penetration Controlled release in the body	Enhance delivery to intestinal/gastric mucosa and biofilms; Enhance permeation of terpenoids into EPS/mucoid functions	[189]
Lipid‐based nanoformulations	Thymol‐, limonene‐liposomes, UA	Liposomes and nanostructured lipid carriers	Regulate release kinetics Enhance uptake throughout epithelial barriers	Enhanced oral absorption Controlled release dynamics within the gastrointestinal tract Deeper diffusion into the biofilm's matrix	[204]
Biopolymer nano‐enabled films and coatings	Thymol, limonene, UA	Alginate + thymol‐NLC; polysaccharide nano‐emulsions	Edible films with embedded NLC enhanced controlled delivery and release dynamics	Enhance antimicrobial activity and potential mucosal biofilm interaction	[190, 205]
Nano‐aided oral targeted carriers	Cell‐membrane mimetic NPs; mucus‐penetrating NPs; lectin‐modified NPs	UA‐lectin NPs, limonene‐mucoadhesive NPs, thymol‐orodispersible films	Mucus‐ and epithelium‐inspired nanosurfaces prolong residence time Improving oral bioavailability and precise drug administration	Utilize biological adhesion and penetration mechanisms to enhance interaction with gut and oral biofilms and improve oncological delivery.	[206]

Nanocarrier systems, such as liposomes, micelles, dendrimers, solid lipid nanoparticles, inorganic nanoparticles, or carbon nanotubes, enable targeted delivery of terpenoids to tumor tissues, primarily due to the permeability and retention effect caused by the lack of adequate lymphatic drainage (Figure 12) [207]. Liposomes and polymeric nanoparticles act as promising carriers for solid tumors like breast, lung, and ovarian cancers due to their ability to solubilize the drug or improve the sustained release property (Table 4) [194]. Ethosomes or phytosomes are favored for cutaneous or hepatic distribution; inorganic nanoparticles are more beneficial for medicinal applications [194]. With the therapeutic potential encased in nanosized particles, the efficiency of the former increased for regulated release with reduced cytotoxicity (Table 4) [194, 203]. These nanoformulations induce apoptosis in cancer cells through mitochondrial damage and caspase activation, in addition to causing lasting inhibition of the cell cycle, hence interfering with the process of autophagy, which is critical for the sustenance of life (Figure 12). It enables better targeting of the tumor with better retention due to the permeability and retention effect (EPR) in the tumor tissues (Figure 12) [208].

### 6.1 Regulatory Considerations and Translational Roadmap for Nano‐Enabled Dietary Terpenoids

Studies indicate that dietary terpenoids formulated into nanotechnology exhibit significant antibiofilm and anticancer properties, but the therapeutics and food sector translation of these compounds needs to be thoroughly investigated through regulatory testing, scaling up of the production process, and comprehensive safety analysis [209]. Nanoscale properties of the nano‐enabled formulation may affect gastrointestinal absorption, tissue distribution, and toxicity, and detailed characterization of the particle size, surface chemistry, stability, biodistribution, and biological interactions is required, in contrast to the more traditional phytochemicals. Physicochemical characterization, exposure assessment, gastrointestinal fate, and repeated‐dose toxicological studies are highlighted before approval by regulatory bodies like the US FDA and the European EFSA for engineered nanomaterials in functional food applications [210, 211]. In addition, Good Manufacturing Practice (GMP) compliant manufacturing, batch‐to‐batch repeatability, pharmacokinetic studies, nanotoxicology studies, immunogenicity and long‐term safety evaluations are some of the requirements for therapeutic translation. Importantly, the nanoformulations should show clinical improvements in bioavailability and clinically meaningful improvements in therapeutic efficacy, which are beyond just improved physicochemical parameters [209, 212, 213]. Validation in the future should use clinically relevant polymicrobial biofilm models, patient‐derived organoids, gut‐on‐chip devices and systems with integration of the microbiome, and use AI‐driven multi‐omics for patient stratification. Moreover, scaling up manufacture to meet Quality‐by‐Design requirements and the harmonization of international regulatory requirements will be necessary to move nano‐enabled dietary terpenoids from laboratory to precision nutrition and complementary cancer therapies [14, 21, 214].

## 7 Future Research Perspectives

Biofilm‐associated microorganisms contribute to tumorigenesis and promote inflammation, immune evasion, chemoresistance, and more, with the strongest correlation found in gastric and CRCs [15]. Food‐based terpenoids are promising dual‐function agents that can disrupt microbial signaling (quorum sensing), EPS matrices, and virulence gene expression while also dampening microbial‐driven oncogenic signaling [35, 84]. However, these models are misaligned with the current lack of internationally accepted models combining antibiofilm and cancerous elements, the scant microbiome‐informed nutritional oncology trials, and the still unclear GRAS and other regulatory issues [215]. Taking into account the microbiome factors and tumor biofilm measurements, as well as the dietary exposure to terpenoids, may help clarify the potential anticancer activity and regulatory effects of the terpenoids on the biofilm (Table 5). With the increased awareness of the complex interactions of diet, the microbiome, and cancer, biologically active dietary factors, such as terpenoids, hold a promising place in the prevention and complementary management of cancer (Table 5) [53].

**Table 5:** Existing gaps and proposed solutions for improving terpenoid crosstalk in biofilm‐driven cancer activity.

Existing gaps	Proposed solution	Performing models	References
Combined antibiofilm–cancer paradigms	Coculture with spatially resolved practices	Organoids/3D tumor‐biofilm prototypes and novel delivery system approach	[216, 217, 218]
Lack of correlational study between diet, microbiome and tumor growth/suppression	Clinical study on diet‐microbiome	Dietary intake markers; metabolomics together with microbiome genetic sequencing	[215, 218]
Lack of regulatory standards	Optimum dose formulation and safety regulation	Toxicology studies and regulatory science (GRAS vs. therapeutic)	[172, 215, 219]
Inadequate mechanistic framework of terpenoids (compound‐specific) with antibiofilm activity	Mapping of QS‐EPS‐virulence signaling pathways with compound‐specific terpenoids	Application of multi‐omics	[84, 220, 221]
Lack of taxonomy mapping cancer types with biofilm‐forming microbial taxa	Biofilm‐forming microbiome–tumor profiling	Application of genomics and biofilm phenotyping	[8, 217, 218]

## Author Contributions

Saikat Mazumder and Aditi Dey contributed equally. Debasmita Bhattacharya, Dibyajit Lahiri, Moupriya Nag, and Soumya Pandit conceptualized and supervised. Saikat Mazumder, Navin Kumar, and Vaseem Raja edited and reviewed. Shubham Sharma, Shashi Prakash Dwivedi, Mithul Rajeev, Juwita Ratna Dewi, Lola Sanayeva, and Dilbar Azimova contributed to resources and visualization.

## Funding

The authors have nothing to report.

## Conflicts of Interest

The authors declare no conflicts of interest.

## Data Availability

Data sharing is not applicable to this article as no data sets were generated or analyzed during the current study.
